# Withanolide E sensitizes renal carcinoma cells to TRAIL-induced apoptosis by increasing cFLIP degradation

**DOI:** 10.1038/cddis.2015.38

**Published:** 2015-02-26

**Authors:** C J Henrich, A D Brooks, K L Erickson, C L Thomas, H R Bokesch, P Tewary, C R Thompson, R J Pompei, K R Gustafson, J B McMahon, T J Sayers

**Affiliations:** 1 Molecular Targets Laboratory, NCI-Frederick, Frederick, MD, USA; 2 Basic Research Program, Leidos Biomedical Research, Inc., Frederick National Laboratory for Cancer Research, Frederick, MD, USA; 3 Laboratory for Experimental Immunology and Cancer Inflammation Program, NCI-Frederick, Frederick, MD, USA; 4 Department of Chemistry, Clark University, Worcester, MA, USA

## Abstract

Withanolide E, a steroidal lactone from *Physalis peruviana*, was found to be highly active for sensitizing renal carcinoma cells and a number of other human cancer cells to tumor necrosis factor-related apoptosis-inducing ligand (TRAIL)-mediated apoptosis. Withanolide E, the most potent and least toxic of five TRAIL-sensitizing withanolides identified, enhanced death receptor-mediated apoptotic signaling by a rapid decline in the levels of cFLIP proteins. Other mechanisms by which TRAIL sensitizers have been reported to work: generation of reactive oxygen species (ROS), changes in pro-and antiapoptotic protein expression, death receptor upregulation, activation of intrinsic (mitochondrial) apoptotic pathways, ER stress, and proteasomal inhibition proved to be irrelevant to withanolide E activity. Loss of cFLIP proteins was not due to changes in expression, but rather destabilization and/or aggregation, suggesting impairment of chaperone proteins leading to degradation. Indeed, withanolide E treatment altered the stability of a number of HSP90 client proteins, but with greater apparent specificity than the well-known HSP90 inhibitor geldanamycin. As cFLIP has been reported to be an HSP90 client, this provides a potentially novel mechanism for sensitizing cells to TRAIL. Sensitization of human renal carcinoma cells to TRAIL-induced apoptosis by withanolide E and its lack of toxicity were confirmed in animal studies. Owing to its novel activity, withanolide E is a promising reagent for the analysis of mechanisms of TRAIL resistance, for understanding HSP90 function, and for further therapeutic development. In marked contrast to bortezomib, among the best currently available TRAIL sensitizers, withanolide E's more specific mechanism of action suggests minimal toxic side effects.

The use of tumor necrosis factor-related apoptosis-inducing ligand (TRAIL) in cancer therapy has long been an attractive goal given its reported ability to induce apoptosis in cancer cells but not normal cells in a variety of oncologic malignancies.^1, 2, 3, 4^ Unfortunately, TRAIL has recently been reported to have other, non-apoptotic signaling properties,^5^ and the development of TRAIL resistance is common in many types of cancer.^1, 2, 3, 4, 6^ Bortezomib and other proteasome inhibitors may be the best agents currently available for reversing resistance to TRAIL,^1, 4, 7, 8^ but bortezomib has long been known to be quite toxic.^1, 4, 7, 8, 9^ Thus, the search for compounds able to sensitize otherwise resistant cancer cells to TRAIL-induced apoptosis has accelerated in recent years.^1, 2, 3, 4, 6, 10^ High-throughput screening of natural product libraries^11^ followed by isolation and characterization of active components has yielded a number of natural products able to sensitize resistant cancer cells to TRAIL.^12, 13^ A series of TRAIL-sensitizing withanolides from the indigenous South American plant *Physalis peruviana* have now been identified, including four not previously identified as TRAIL sensitizers. Withanolides comprise a family of C_28_ plant steroids many of which induce apoptosis in several tumor cell lines.^14, 15^ A number of mechanisms of action identified for TRAIL sensitizers,^1, 6, 8, 10, 16, 17^ including generation of reactive oxygen species (ROS), induction of ER stress, inhibition of heat shock proteins, proteasomal inhibition and more general effects on transcription, translation, and signal transduction, have also been reported as possible mechanisms for the antitumor cell activities of withanolides.^18, 19, 20, 21, 22, 23, 24, 25, 26, 27^ Withanolide E (the most active and abundant TRAIL-sensitizing withanolide isolated in this work) synergizes with TRAIL to induce cancer cell apoptosis *in vitro* and *in vivo* via enhanced death receptor-mediated TRAIL signaling. Its mechanism of action, cFLIP degradation, appears to be mediated by specific modulation of HSP90 function.

## Results

### Withanolides are TRAIL-sensitizing constituents of an active natural product extract

**Figure 1:**
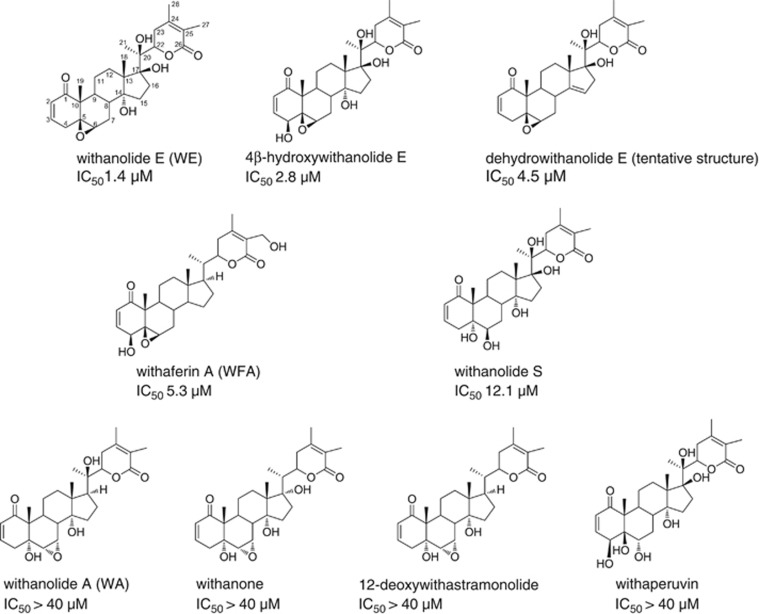
Withanolides vary in their ability to sensitize ACHN cells to TRAIL. IC_50_ values (average, *n*=2–6) were calculated from dose–response data and indicate 50% reduction in cell number by withanolide+TRAIL compared with untreated cells

The most abundant and highest potency withanolide purified and characterized from the *P. peruviana* extract was withanolide E. The structures and activities of this and several other withanolides as TRAIL sensitizers are shown in Figure 1. Withanolide A, withanone, withaperuvin, and 12-deoxywithastramonolide were inactive (up to 40 *μ*M) with or without TRAIL. Withanolide E had the least growth inhibitory effect as a single agent (5.7% reduction in cell number at 10 *μ*M as compared with 10.9% for 4*β*-hydroxy withanolide E, 21.9% for withanolide S, 39.5% for dehydrowithanolide E, and 44.0% for withaferin A).

**Figure 2:**
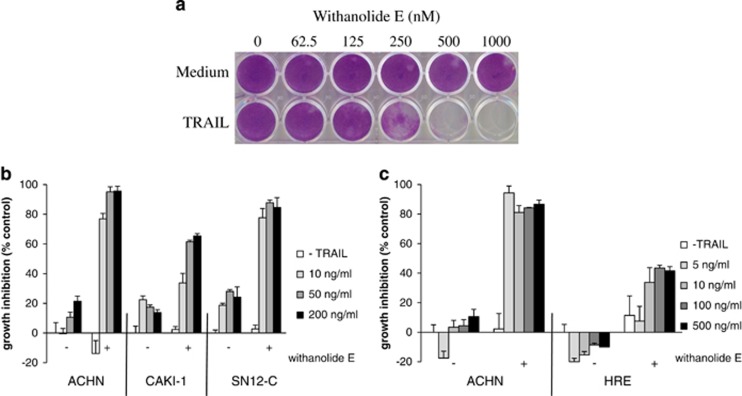
Effects of withanolide E on renal carcinoma cells. (**a**) ACHN cells were treated 4 h with withanolide E, then TRAIL overnight followed by washing and medium replacement. After 5 days, cells were fixed, stained, and photographed. (**b** and **c**) Renal carcinoma (ACHN, CAKI-1, SN12-C) or human renal epithelial (HRE) cells in 96-well plates were treated for 4 h with 1 *μ*M withanolide E followed by 24 h with the indicated concentrations of TRAIL and relative cell numbers assessed. Error bars represent S.D. (*n*=3)

The effects of withanolide E on growth of renal carcinoma cells are shown in Figure 2. Figure 2a clearly demonstrates that withanolide E eliminates long-term survival of ACHN cells, but only in the presence of TRAIL, thus confirming that the combination leads to cell death rather than simply growth inhibition. The increased apparent potency of withanolide E+TRAIL in longer-term experiments suggests that these cells are irreversibly committed to cell death (i.e., they do not subsequently recover upon removal of withanolide E and TRAIL) even though they may be detectable by 2,3-Bis(2-methoxy-4-nitro-5-sulfophenyl)-5-[(phenylamino)carbonyl]-2H-tetrazolium hydroxide (XTT), MTS, or ATP assays after shorter duration (1 day) treatment. Withanolide E was also a very effective TRAIL sensitizer for two other renal carcinoma lines (Figure 2b), but only modestly affected untransformed human renal epithelial (HRE) cells, even at very high TRAIL concentrations (Figure 2c). Similarly, withanolide E alone had minimal effects on the growth of 22 other diverse cancer cell lines, but several TRAIL-resistant cancer cell lines became TRAIL-sensitive after withanolide E treatment (see Supplementary Information). Withanolide E also had only minor effects on morphology of ACHN and CAKI cells (see Supplementary Information).

### Withanolide E enhances normal death receptor signaling

**Figure 3:**
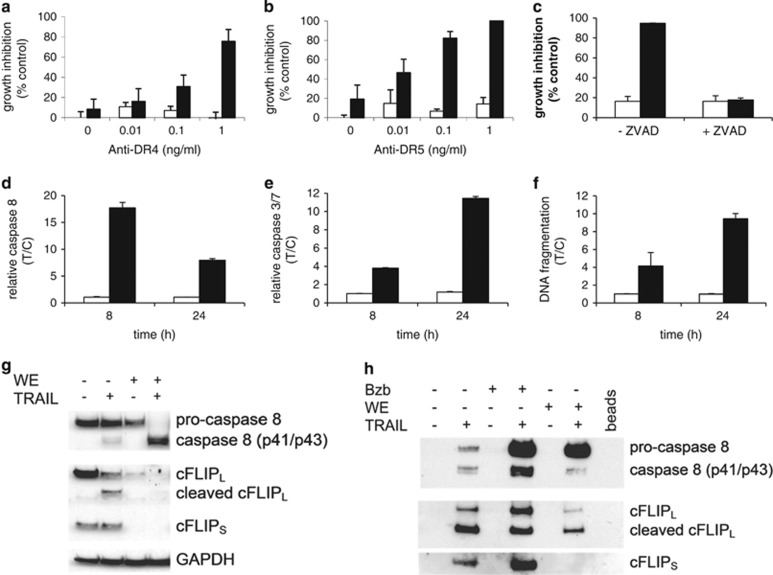
Withanolide E enhances TRAIL-induced extrinsic apoptotic pathway in ACHN cells. Cells were treated without (open bars) or with (filled bars) withanolide E for 3–4 h followed by agonistic anti-DR4 (**a**) or DR5 (**b**) antibodies for 20–24 h. For panels **c**–**f**, cells were treated 4 h±withanolide E (WE) then 4 h (for a total of 8 h) or 24 h±TRAIL in the continued presence of WE. Open bars: withanolide E only; black: withanolide E followed by TRAIL. (**c**) Cells were pretreated with ZVAD-FMK to block caspase activation followed by WE, then TRAIL. Caspase 8 (**d**) and caspase 3 (**e**) activities, and DNA fragmentation (**f**) were assessed using commercial kits. (**g**) Caspase cleavage and cFLIP degradation were assessed by immunoblot (total treatment time, 8 h). (**h**) TRAIL-dependent DISC formation was assessed in withanolide E- or bortezomib (Bzb – control with known activity)-treated ACHN cells (overnight treatment followed by biotinylated TRAIL at 4 °C, cell lysis, DISC precipitation and immunoblot). Error bars represent S.D. (*n*=3)

To assess whether sensitization of ACHN cells to TRAIL by withanolide E occurs via activation of the extrinsic (i.e., TRAIL-dependent) apoptosis pathway, several components of 'normal' TRAIL signaling were measured (Figure 3). Withanolide E sensitized ACHN cells to apoptosis induced by agonistic antibodies to death receptors 4 and 5; thus, indicating that it affected death receptor activation (Figures 3a and b). Sensitization of ACHN cells to TRAIL-induced apoptosis required caspase activation (blocked by ZVAD-FMK, a caspase inhibitor – Figure 3c). Withanolide E enhanced the TRAIL-dependent sequential activation of caspase 8 (>15-fold increase after 4 h followed by 4 h TRAIL – Figure 3d, confirmed by immunoblot – Figure 3g) and caspase 3 (>10-fold increase after 24 h – Figure 3e), followed by DNA fragmentation (Figure 3f), indicative of end-stage apoptosis. Withanolide E alone had minor effects on each of these measurements.

However, withanolide E alone significantly reduced the levels of cellular cFLIP (Figure 3g). TRAIL-dependent death-inducing signaling complex (DISC) formation was robust in the presence of withanolide E, albeit with decreased cFLIP and led to enhanced caspase 8 recruitment, resulting in an increase in the caspase 8:cFLIP ratio (Figure 3h). The control for this experiment, bortezomib, induced greater TRAIL-dependent caspase 8 recruitment than did withanolide E, albeit with greater DISC-associated cFLIP as well. The significantly reduced level of cFLIP in the DISC following withanolide E treatment would suggest increased DISC-associated caspase 8 activation, leading to amplification of TRAIL-induced apoptotic signaling. Together these results confirm that treatment with withanolide E+TRAIL rapidly and irreversibly commits ACHN cells to apoptosis via a rapid drop in the total cellular levels of cFLIP and amplification of the apoptotic signaling from the DISC.

### Effects of withanolide E on mechanisms of TRAIL sensitization

**Figure 4:**
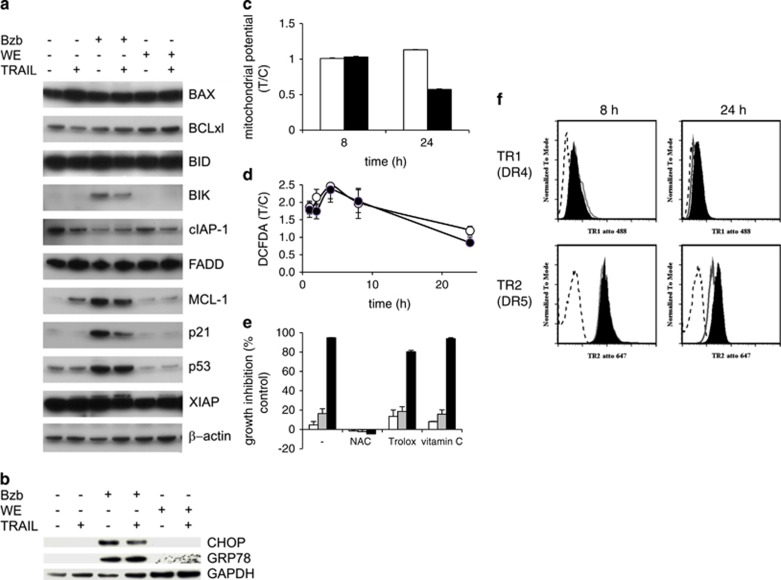
Withanolide E effects on TRAIL sensitization mechanisms. (**a**) Effects of withanolide E on expression of pro- and antiapoptotic proteins. ACHN cells were treated 24 h with bortezomib (20 nM) or withanolide E, then±TRAIL (50 ng/ml) and immunoblot. (**b**) ACHN cells were treated±bortezomib (Bzb, positive control) or withanolide E (WE) followed by±TRAIL (total treatment time, 8 h) and immunoblot analysis of ER stress markers. (**c**) Cells were treated 4 h±withanolide E then 4 h (for a total of 8 h) or 24 h±TRAIL in the continued presence of WE. Open bars: withanolide E only; black: withanolide E followed by TRAIL. Mitochondrial potential was assessed (JC-1 assay). (**d**) Cells were treated with withanolide E (solid symbols) or withanolide A (inactive control, open symbols) and ROS generation estimated with DCFDA. (**e**) ACHN cells were pretreated with NAC (10 mM), Trolox (200 *μ*M) or vitamin C (vitC, 200 *μ*M) followed by withanolide E (2 h), then TRAIL (24 h). Open bars: withanolide E only; gray bars: TRAIL only; black bars: withanolide E+TRAIL. (**f**) ACHN cells were treated for 8 h or 24 h with 10 *μ*M withanolide E and assessed for expression of TRAIL receptors 1 and 2 by FACS (FACS Caliber, BD Biosciences). Antibodies used were (anti-TRAIL-R1 (human), mAb (HS101) (ATTO 488) or anti-TRAIL-R2 (human), mAb (HS201) (ATTO 647N) or mouse IgG1 atto488 or mouse IgG1atto 647 (Adipogen)). Dashed line represents isotype control, solid line DMSO-treated cells, and filled line withanolide E-treated cells. Error bars represent S.D. (*n*=3–4)

Sensitization of TRAIL-resistant cells to TRAIL-induced apoptosis can occur via a variety of mechanisms and effects,^6, 7, 8, 9, 10, 28^ including changes in the levels of pro- and antiapoptotic proteins; ER stress; activation of the intrinsic (mitochondrial) pathway; generation of ROS; death receptor upregulation; proteasome inhibition; DISC dysregulation; caspase activation; cFLIP downregulation. In marked contrast to bortezomib, expression of a panel of proteins previously reported to be important for intrinsic apoptosis signaling and TRAIL-driven apoptosis was not significantly affected by withanolide E alone (Figure 4a and Supplementary Information), and indicators of ER stress (CHOP and GRP78) were unaffected by withanolide E (Figure 4b). Similarly, withanolide E does not induce mitochondrial depolarization except in the presence of TRAIL (i.e., late-stage apoptosis – Figure 4c). By contrast, doxorubicin was previously identified as a TRAIL sensitizer in the initial screen,^11^ and it activates the intrinsic mitochondrial pathway^29^ as demonstrated by induction of mitochondrial depolarization (data not shown), thus confirming that the ACHN cells utilized here can be sensitized to TRAIL, at least in part, via induction of the intrinsic apoptotic pathway. Both withanolide E and the inactive withanolide A induced transient increases in ROS, peaking at 4 h, but by 24 h there was no longer a measurable ROS increase (Figure 4d). However, pretreatment with withanolide E for 24 h (i.e., after ROS decayed) followed by short incubation with TRAIL resulted in complete cell killing (data not shown). The presence of TRAIL had no effect on withanolide-induced ROS generation (data not shown). NAC treatment eliminated the cell killing effect of withanolide E in the presence of TRAIL, but two other antioxidants (vitamin C and Trolox) had no such effect (Figure 4e). Consistent with previous reports of reactivity between withanolides and NAC,^30^ NAC formed a covalent chemical adduct with withanolide E (see Supplementary Information). Thus, ROS generation is unlikely to contribute to the TRAIL-sensitizing activity of withanolide E and chemical reactivity rather than ROS quenching explains NAC's inhibitory activity. Death receptor (DR4 and DR5) expression was only modestly affected by withanolide E (Figure 4f), in contrast to previously reported induction of increased DR4 and DR5 expression by the TRAIL sensitizer bortezomib.^31^ Withanolide E treatment did not lead to inhibition of proteasomal activity or accumulation of ubiquitinated proteins (see Supplementary Information) nor did it affect the levels of caspases (Figure 3).

### Withanolide E induces cFLIP degradation

**Figure 5:**
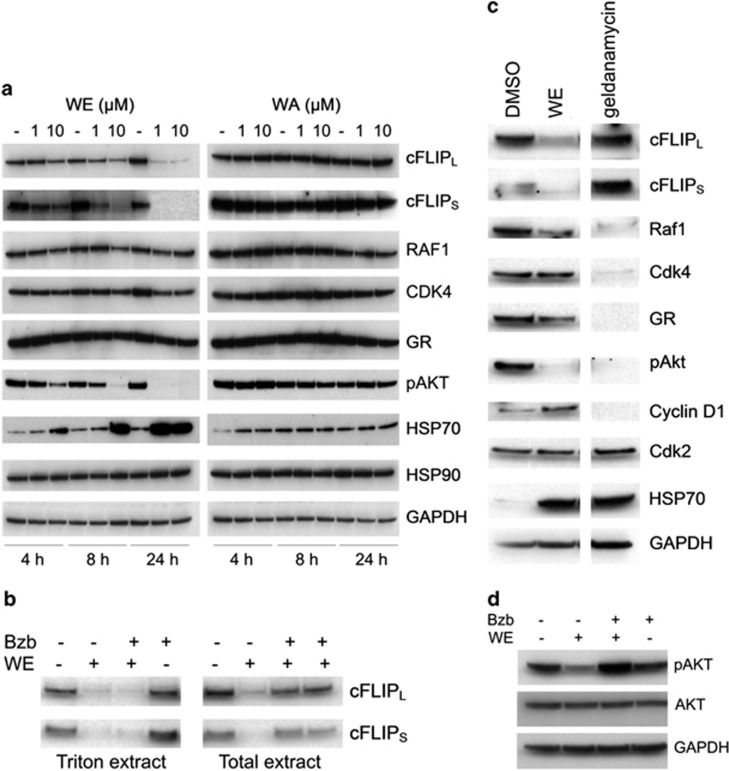
Withanolide E affects cFLIP and HSP90 client proteins. (**a**) Cells were treated for 4–24 h with 1 or 10 *μ*M withanolide E (WE) or withanolide A (WA) followed by immunoblot detection of indicated proteins. (**b**) Cells were treated±WE±bortezomib (40 nM) and processed by extraction with a Triton X-100-containing extraction buffer or with SDS-containing sample buffer to yield 'Triton ext' and 'Total ext' samples, respectively, and analyzed for cFLIP by immunoblot. (**c**) Cells were treated for 8 h with geldanamycin (positive control) or WE followed by immunoblot. (**d**) ACHN cells were treated±WE±bortezomib (Bzb) followed by immunoblot analysis of pAKT and total AKT

Of the TRAIL-sensitization mechanisms assessed, only withanolide E-induced cFLIP downregulation was observed, specifically rapid reduction in total cellular cFLIP (Figure 3g) without affecting caspase 8 or FADD (Figures 3g and 4a). TRAIL-dependent caspase activation was not required for cFLIP downregulation, but residual cFLIP_L_ was completely eliminated on subsequent addition of TRAIL (Figure 3g). Significant reduction of cFLIP was seen as early as 4 h after treatment with as low as 1 *μ*M withanolide E and by 24 h, the short form was undetectable and the long form barely detectable (Figure 5a). Downregulation of cFLIP protein can occur rapidly upon blocking cFLIP mRNA transcription.^32, 33, 34, 35, 36^ However, withanolide E treatment did not have a significant effect on cFLIP mRNA levels (see Supplementary Information), making it likely that the drop in cFLIP is due to increased degradation. cFLIP reduction could result from dysregulation/aggregation, first leading to altered subcellular distribution and solubility and, ultimately, degradation. When proteasomal activity was inhibited by bortezomib, withanolide E-induced degradation of cFLIP was blocked and cFLIP accumulated, but cFLIP redistributed to a Triton-insoluble cellular compartment (Figure 5b). Bortezomib alone does not have this effect. These results suggest withanolide E-induced misfolding and/or aggregation of cFLIP. This, together with the reported effects of other withanolides on heat shock proteins^19, 37^ and reports that cFLIP is an HSP90 client protein,^38^ led to an investigation of the effects of withanolide E on HSP90 client proteins. The inactive withanolide A served as a negative control, and it elicited no changes in any of the proteins assessed (Figure 5a). HSP70 expression was increased by withanolide E and by geldanamycin, (Figures 5a and c), consistent with inhibition of HSP90,^39^ but neither compound affected levels of CDK2, a protein not chaperoned by HSP90 (Figures 5a and c). Several other established HSP90 client proteins, RAF1, CDK4, glucocorticoid receptor, and cyclin D1, were downregulated by geldanamycin but were unaffected or only modestly affected by withanolide E (Figure 5c). Both withanolide E and geldanamycin reduced pAKT levels. By contrast, only withanolide E resulted in reduced cFLIP levels (Figure 5c). Geldanamycin did not efficiently sensitize ACHN cells to TRAIL (data not shown). The withanolide E effects on pAKT, HSP70, and cFLIP all had similar time- and dose-dependencies, but expression of HSP90 itself was unaffected (Figure 5a). Inhibition of proteasomal activity by bortezomib blocked the withanolide E-induced degradation of pAKT without affecting total AKT (Figure 5d), suggesting that withanolide E affects pAKT protein stability. It has been reported that HSP90 can preferentially stabilize activated forms of protein kinases (for example, Met^40^). Thus, it is reasonable to conclude that the loss of pAKT as a result of withanolide E treatment is HSP90-related rather than a result of changes in phosphorylation/dephosphorylation. These results also suggest that HSP90 inhibition may only generate TRAIL sensitization when it leads to cFLIP degradation. Inclusion of withaferin A, known to bind to HSP90,^19, 37^ at a sub-sensitizing/sub-toxic concentration (0.5 *μ*M) completely eliminated the TRAIL-sensitizing activity of withanolide E (<5% growth inhibition in the presence of withanolide E+withaferin A+TRAIL).

### *In vivo* enhancement of TRAIL-induced antitumor effects by withanolide E

**Figure 6:**
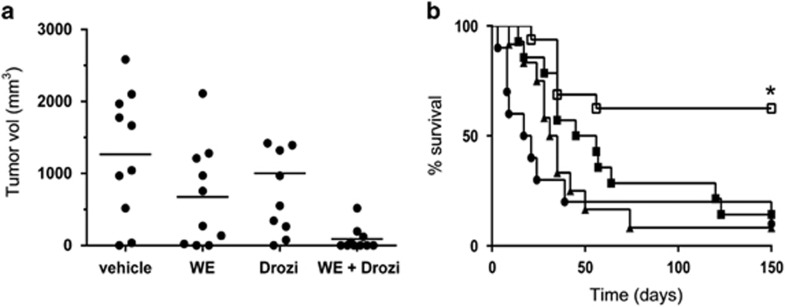
Withanolide E enhances death receptor-induced apoptosis *in vivo*. Effects of withanolide E and drozitumab (DR5 agonist antibody) on growth of ACHN cell-derived tumors in athymic mice was assessed as described in the text. (**a**) Tumor size at 75 days (intratumor), (**b**) survival up to 150 days (intraperitoneal) – pooled from two separate experiments with similar findings. Numbers of mice were: vehicle control (●, *n*=10), withanolide E (▴, *n*=10), drozitumab (▪, *n*=14), withanolide E plus drozitumab (□, *n*=16). *P<0.05

As withanolide E promoted TRAIL-induced apoptosis in ACHN cells *in vitro*, the combination of withanolide E and an agonist antibody to TRAIL death receptor DR5 was assessed *in vivo*. Figure 6 demonstrates the *in vivo* efficacy of withanolide E as a TRAIL sensitizer in a mouse model system. Intra-tumor administration of the combination of withanolide E and drozitumab (DR5 agonistic antibody) was more effective in decreasing tumor progression than either agent alone. Intraperitoneal administration of the combination provided a superior therapeutic benefit over either agent alone in long-term tumor survival studies. The combination of agents resulted in >55% of the mice having no detectable palpable tumor 150 days after the start of therapy. A further prolonged follow-up of some of these surviving mice for over 250 days showed no further signs of tumor, consistent with a complete and sustained tumor regression in these individuals. The animals were monitored for indicators of overt toxicities and were weighed twice weekly. No obvious toxicities were observed at any stage during the administration of these treatment schedules (data not shown).

## Discussion

Withanolide E was the most effective TRAIL sensitizer, but least toxic, of the withanolides assessed. Withanolide E enhanced the extrinsic/death receptor-dependent apoptotic pathway rather the intrinsic mitochondrial pathway and, as a single agent, had no significant pro-apoptotic effects. Its most significant effect was a rapid decrease in cellular cFLIP. cFLIP is a major regulator of TRAIL signaling,^33, 41, 42^ and loss of cFLIP can lead to increased TRAIL-induced apoptosis.^34, 35, 36^ Rapid loss of cFLIP protein and only minor changes in cFLIP mRNA suggest increased degradation in response to withanolide E. Blocking degradation drove residual cFLIP into a less soluble cellular compartment consistent with denaturation or aggregation. Thus cFLIP dysregulation may prime cells for response to death receptor activation. Recent reports have demonstrated that cFLIP has a critical role in tissue homeostasis by preventing apoptosis and necroptosis. Conditional knockout of cFLIP in skin,^43^ intestinal epithelia,^44^ or liver^45, 46^ resulted in cell death in response to endogenous death ligands. cFLIP is essential in preventing inappropriate cell death in normal tissues, suggesting that a reduction in cFLIP may increase apoptosis in neoplastic disease. Given the literature reports of other potential mechanisms of action for TRAIL sensitizers, characterization of the TRAIL-sensitizing activity of withanolide E included assessment of effects on apoptotic protein expression, proteasomal activity, generation of ROS, ER and mitochondrial stress, and death receptor expression. As demonstrated in the results section and cited references, each of these mechanisms can be activated in the present experimental system but withanolide E does not utilize them to sensitize cells to TRAIL. ROS generation was not a factor in the TRAIL-sensitizing activity of withanolide E, and withanolide E had little effect on the other phenomena. Interestingly, one other withanolide, withaferin A, has been previously identified as a TRAIL sensitizer.^47^ In contrast to withanolide E, withaferin A did not significantly affect cFLIP. However, consistent with previous reported targets,^48^ withaferin A had significant effects on ER stress and on cellular morphology and inhibited proteasomal activity (data not shown and Supplementary Information) and it was much more toxic to ACHN cells as a single agent than was withanolide E. Critically, siRNA-mediated reduction of cFLIP results in sensitization of renal carcinoma cells to TRAIL-mediated apoptosis,^34^ demonstrating that the reduction in cFLIP alone is sufficient to sensitize these cells. Thus, cFLIP dysregulation was the only relevant TRAIL-sensitizing activity observed for withanolide E.

Heat shock proteins, particularly HSP90, have been reported to mediate some cellular responses to withanolides.^18, 19, 37^ Withanolides may interact with and destabilize HSP90, with a resultant elimination of client proteins via the proteasome. It has previously been reported that siRNA-mediated reduction of HSP90 sensitized glioblastoma cells to TRAIL apoptosis by effects on cFLIP, suggesting that cFLIP is an HSP90 client protein and that reduced levels of HSP90 prevented appropriate trafficking of cFLIP to the TRAIL DISC.^38^ Thus, the effects of withanolide E are consistent with HSP90 inhibition leading to misfolding and/or aggregation of cFLIP culminating in cFLIP degradation. Redistribution of cFLIP to less soluble (i.e., Triton-insoluble) cellular fraction(s) is consistent with this interpretation. Treatment of ACHN cells with withanolide E clearly affected the levels of some HSP90 client proteins, specifically cFLIP and phosphoAKT. Inhibitors of AKT activation did not sensitize ACHN cells to TRAIL (data not shown). Thus loss of phosphoAKT is probably a result of HSP90 inhibition by withanolide E, but not a driver of withanolide E-induced cFLIP downregulation or TRAIL sensitization in these cells. In contrast to geldanamycin, withanolides (including withanolide E) have been reported to bind to the C-terminal domain of HSP90.^19^ This may account in part for the greater selectivity of withanolide E with regard to HSP90 inhibition and the spectrum of client proteins affected. Withaferin A, a known HSP90-binding compound, blocked the TRAIL-sensitizing effect of withanolide E. Its ability to block withanolide E is consistent with the suggestion that the TRAIL-sensitizing effect of withanolide E may be HSP90-dependent and that the two compounds compete for the same or nearby binding site(s) and/or that withaferin A induces a conformational change such that withanolide E is no longer able to bind. Preliminary results suggest that withanolide E can bind to HSP90 and follow-up experiments are underway to identify the nature of this interaction as well as to investigate other mechanisms of cFLIP downregulation.

Insufficient numbers of withanolides have been assessed as TRAIL sensitizers to develop significant structure-activity relationships (SAR), but small structural differences clearly lead to significant diversity in activity. However, the cytotoxicity/apoptosis-inducing activity of withanolides has been the subject of several SAR studies. The C2,C3 double bond in ring A is absolutely required and the C5,C6 epoxide moiety enhances toxicity, whereas withanolides with a C6,C7 epoxide are relatively innocuous.^18, 19, 20, 30, 49, 50, 51^ As shown in this work (Figure 1), withanolides with the C5,C6 epoxide were active as TRAIL sensitizers, whereas those with the C6,C7 epoxide were inactive. The C4 hydroxyl enhances toxicity in many cell types,^18, 19, 50^ but had little, if any, effect on sensitization of ACHN cells to TRAIL (compare withanolide E and 4*β*-hydroxy withanolide E). It has been reported that the C17 and C20 hydroxyls may slightly reduce withanolide toxicity.^49^ These structural features generally correlate with the toxicity of the compounds in the absence of TRAIL as seen above. SAR for cytotoxicity/apoptosis and for HSP90 effects are also correlated.^18, 19, 30^ Recent studies demonstrated that a hydroxyl at C4 affects reactivity with HSP90 and specificity for other targets. Withaferin A and 4*β*-hydroxy withanolide E were reported to disrupt the interaction of HSP90 with CDC37, but withanolide E (lacking the C4 hydroxyl) was much less effective.^19^ Thus the structural features that differ between withanolide E and the significantly more toxic (and less potent as a TRAIL sensitizer) withaferin A may result in differential specificity for HSP90 and client proteins, including cFLIP. Additional experiments are underway to better characterize the interactions between TRAIL-sensitizing withanolides and HSP90 proteins and may contribute to future design of low toxicity HSP90-targeted agents.

A number of human cancer cells were sensitized to TRAIL-induced apoptosis by withanolide E, whereas others remained resistant. The molecular basis for this difference is unknown and is being investigated but may be related to their relative expression of cFLIP. For example, cFLIP is reported to have a central role in conferring TRAIL resistance to melanoma^52, 53^ and glioblastoma,^54, 55^ and several TRAIL-resistant melanoma and glioblastoma cell lines were sensitized by withanolide E to TRAIL-induced apoptosis (see Supplementary Information). Thus, withanolide E may prove to be a powerful reagent for assessing the relative importance of cFLIP levels for TRAIL resistance in cancer cells, as well as providing a means of overcoming cFLIP-associated TRAIL resistance. The effects of withanolide E on cFLIP protein stability rather than transcription may also allow for greater specificity and more focused therapeutic applications. The identification of withanolide E as a novel and selective modifier of HSP90:client protein interactions will provide a better understanding of the role of HSP90 in TRAIL signaling. The contrast between withanolide E and bortezomib, among the best available TRAIL sensitizers, is also significant. Bortezomib is well-known to exhibit *in vitro* and *in vivo* toxicity, possibly because of the global effects of inhibiting the proteasome and its potency as a general cell stressor. On the other hand, withanolide E has minimal effects on multiple mechanisms (e.g., cell stress, mitochondrial effects, ROS, etc.) that could lead to significant toxicity and/or other side effects. Its lack of toxicity as a single agent *in vitro* and *in vivo* as well as its limited ability to induce apoptosis in normal cells at modest TRAIL concentrations bode well for its potential therapeutic utility.

Thus withanolide E may prove to be a powerful reagent for increasing understanding of HSP90 function as well as mechanisms of cellular resistance to TRAIL-induced apoptosis, and may have future therapeutic application in combination with the targeting of death receptor signaling in cancer cells.

## Materials and Methods

### Chemicals and reagents

Withanolides were purified from *P. peruviana* extracts (NCI Natural Products Repository) as described in Supplementary Data and/or obtained from the NCI Developmental Therapeutics Program and/or from Chromadex (Irvine, CA, USA). Sources of other reagents were XTT (NSC 601519) from the NCI Drug Synthesis and Chemistry Branch; bortezomib (NIH Pharmacy, Bethesda, MD, USA); recombinant TRAIL ligand (168 amino acid TNF homologous extracellular domain - Peprotech, Rocky Hill, NJ, USA); Z-VAD-FMK (BioMol, Plymouth Meeting, PA, USA); cell culture media and additives (Cellgro (Manasses, VA, USA), Hyclone (Logan, UT, USA), Sigma (St. Louis, MO, USA), or Invitrogen (Carlsbad, CA, USA)); BCA protein assay kits (Pierce/Thermo, Rockford, IL, USA); other reagents from Sigma. Chemical structures were drawn using ChemDraw (CambridgeSoft Corp., Cambridge, MA, USA) using structural information from the PubChem database (http://pubchem.ncbi.nlm.nih.gov/).

### Cell growth assays

ACHN, CAKI-1, and SN12-C cell lines (NCI) and HRE cells were from (Lifeline Cell Technology, Frederick, MD, USA), and were maintained as recommended by source institutions. Growth was assayed as described.^11^ In brief, cells were allowed to attach overnight (3500 cells/well, 384-well or 5000 cells/well, 96-well plates) followed by 2–4 h with compounds or DMSO. TRAIL was added and cell numbers were estimated (24 h) using XTT^11^ or MTS (Promega, Madison, WI, USA). For analysis of ROS involvement, N-acetyl cysteine (NAC, 10 mM), Trolox (200 *μ*M), or vitamin C (200 *μ*M) was added just before compounds and remained in the plates throughout. NAC interfered with XTT and MTS (data not shown), so cell numbers were estimated using a luminescence ATP assay (Promega). For caspase inhibition, cells were pretreated with 100 *μ*M ZVAD-FMK. To assess death receptor utilization, agonistic anti-DR4 (Alexis/Enzo, Farmingdale, NY, USA) or DR5 (R&D Systems, Minneapolis, MN, USA) replaced TRAIL.

Unless otherwise noted, in all experiments, withanolide and TRAIL concentrations were 1 *μ*M and 20–40 ng/ml, respectively.

### Long-term survival of ACHN renal cancer cells

Clonal outgrowth experiments were performed to confirm cell death rather than just growth inhibition.^11^ Withanolide E was added for 3 h, followed by TRAIL (500 ng/ml). The next day, plates were washed and medium replaced. After 5 days, plates were methanol fixed, stained with Crystal Violet, and visualized.

### Immunoblot

ACHN cells (2 × 10^6^ cells/well, 6-well plates) were extracted for SDS-PAGE and immunoblot analysis as described.^7^ In some experiments, cells were lysed with either a Triton X-100-containing extraction buffer or with SDS-containing RIPA buffer (both containing 40 *μ*M ZVAD). Triton supernatants and total cell extracts were subjected to SDS-PAGE and immunoblot. Primary antibodies, detection reagents, and methods are listed in Supplementary Information.

### Apoptotic signaling

ACHN cells (3500 cells/well, 384-well plates) were treated for 4 h followed by TRAIL (4–24 h). Assessment of caspase 8 or 3 utilized Caspase-Glo 8 or 3/7 assay kits (Promega). DNA fragmentation was estimated using the Roche Cell Death ELISA system. Relative mitochondrial potential was assessed using the ratiometric fluorescent dye JC-1.^56^ Cells (6-well plates, 500 000 cells/well) were treated 4 h followed by TRAIL. After 30 min at 37 °C with 2 *μ*M JC-1 (added immediately after dilution), green and red fluorescence data acquired on an BD/Accuri C6 flow cytometer and analyzed using the instrument's CFLOW software (BD/Accuri Cytometers, San Jose, CA, USA). The fluorescence ratio is a measure of mitochondrial potential.^56^

### ROS detection

Generation of ROS was quantitated using DCFDA, a compound that fluoresces upon oxidation by ROS. After cell treatment, CM-H_2_DCFDA (Invitrogen) was added (25 *μ*M) for the last 1 h of incubation. After washing, cell-associated fluorescence was measured on a Tecan fluorometer at Ex 495 nm, Em 529 nm (bottom read mode).

### Immunoprecipitation of DISC

Cells (2 × 10^6^ cells/well, 6-well plates) were treated with 20 nM Bortezomib or 1 *μ*M withanolide E overnight (37 °C) then 2 h (4 °C) with 500 ng/ml biotinylated TRAIL (rhTRAIL (Peprotech) biotinylated using PlatinumLink Labeling Kit, Kreatech Diagnostics (Amsterdam, The Netherlands)). After cold PBS wash, lysis in IP DISC lysis buffer (30 mM Tris-HCL pH 7.5, 150 mM NaCl, 10% glycerol, 1% Triton X-100+protease inhibitors and 20 *μ*M Z-VAD-FMK), and equalization for protein concentration, extracts were incubated with Dynabeads MyOne Strepavidin T1 resin (Invitrogen) (15 min, 4 °C), washed in IP DISC lysis buffer, eluted in 2 × NuPAGE LDS Sample Buffer and NuPAGE Reducing Agent (Invitrogen), and subjected to immunoblot analysis.

### Animal studies

Athymic BALB/c mice were obtained from the Animal Production program (Charles River Laboratories, Inc., Frederick, MD, USA). For the generation of subcutaneous tumors, mice were injected subcutaneously with 1 × 10^6^ ACHN cells that had been adapted for *in vivo* passage in a volume of 100* μ*l. After tumor volumes reached ~100 mm^3^ (~14 days post injection) therapy was initiated - either direct injection of withanolide E and Drozitumab (agonist antibody to TRAIL death receptor DR5, kindly provided by Dr. Avi Ashkenazi, Genentech, South San Francisco, CA, USA^57, 58^) into the tumor, or intraperitoneal injection of both agents. For intratumor injections, withanolide E was administered in PBS/50% DMSO in a volume of 50 *μ*l (20 mg/kg), whereas for intraperitoneal injections, withanolide E was in DMSO in a volume of 20 *μ*l to give a dose of 20 mg/kg. Drozitumab in PBS was injected in a volume of 50 *μ*l for both intratumor and intraperitoneal injections to give a final dose of 5 mg/kg. Control groups were treated with the vehicles alone. The weekly treatment schedule was injection of withanolide E followed the next day with drozitumab, then 1 day pause, followed by a second dose of withanolide E with a second administration of drozitumab 1 day later followed by a 2 days pause. Four weekly cycles of the drugs were given. Mice were continuously monitored for any signs of distress. Two perpendicular diameters of the tumor were measured twice per week using calipers, and the tumor volume was calculated using the formula: *V*=0.5*a* × *b*^2^ with a and b being the long and short diameters of the tumor, respectively. For survival studies, when the tumors reached greater than 500 mm^3^ or if the tumor had been necrotic for more than 4 weeks with no evidence of regression, mice were painlessly euthanized with CO_2_ as outlined in the Frederick National Laboratory for Cancer Research Animal Care and Use Committee (ACUC) guidelines.

### Data presentation and analysis

Results were normalized to control (DMSO)-treated cells. Unless otherwise noted, values reported represent average±S.D. IC_50_ values were estimated from dose–response curves using SigmaPlot 4-parameter logistic nonlinear regression analysis (SPSS, Inc., Chicago, IL, USA). For survival studies Kaplan–Meier plots were generated and *P-*values were obtained with the log-rank Mantel–Cox test (Prism software version 6.0 (GraphPad Software, Inc., LaJolla, CA, USA)).

## Abbreviations

DISC: death-inducing signaling complex
cFLIP: cellular FLICE-like inhibitory protein
NAC: N-acetyl cysteine
ROS: reactive oxygen species
TRAIL: tumor necrosis factor-related apoptosis-inducing ligand
WE: withanolide E
WA: withanolide A
WFA: withaferin A
