# Supplementary material for: Withanolide E sensitizes renal carcinoma cells to TRAIL-induced apoptosis by increasing cFLIP degradation
Source: Cell Death Dis. 2015 Feb 26;6(2):e1666–. doi: 10.1038/cddis.2015.38 (PMC4669816; doi:10.1038/cddis.2015.38)
Supplement: Supplementary Information [file cddis201538x1.pdf]

SUPPLEMENTARY INFORMATION

Henrich, et al., **Withanolide E sensitizes renal carcinoma cells to TRAIL-induced apoptosis by increasing cFLIP degradation**

Withanolide isolation and characterization

The areal portions of the shrub *Physalis peruviana* were collected in Madagascar near the Manakazo River habitat in May 1991. They were collected and identified by Dr. James S. Miller and a voucher specimen (Q66V-7534) is maintained at the Missouri Botanical Gardens, St. Louis, MO. The air dried plant material (251 g) was successively extracted with CH_2Cl_2-MeOH (1:1) and 100% MeOH following the standard NCI plant extraction protocol (McCloud, 2010). Removal of the combined solvents under reduced pressure provided 8.29 g of crude extract (NSC # N038147). A 1.0 g aliquot of this extract was separated on a 60 mL column of diol stationary phase by sequential elution with hexane-CH_2Cl_2 (9:1), CH_2Cl_2-EtOAc (20:1), 100% EtOAc, EtOAc-MeOH (5:1), and 100% MeOH. The material that eluted with 100% EtOAc (185 mg) was then chromatographed on a 200 mL column of Sephadex LH-20 with CH_2Cl_2-MeOH (1:1). The main UV absorbing peak (110 mg) was re-chromatographed on LH-20 eluted with hexane-CH_2Cl_2-MeOH (2:5:1) followed by vacuum liquid chromatography on silica gel eluted with 100% CH_2Cl_2, CH_2Cl_2-EtOAc (1:1), 100% EtOAc, and mixtures of EtOAc-MeOH. Final purification was achieved by silica preparative TLC developed with EtOAc-MeOH (99:1) to give withanolide E (6.9 mg), 4β-hydroxywithanolide E (2.9 mg), 14,15-dehydrowithanolide E (2.2 mg), and withanolide S (1.6 mg).

The structures of the withanolides isolated from *P. peruviana* were assigned by comparison of comprehensive sets of NMR and mass spectrometry data recorded for these compounds with appropriate values in the chemical literature (Lavie, et al., 1972; Kirson, et al., 1976; Sakurai, et al., 1976; Glotter, et al., 1977). Withanolide E was also verified by comparison with an authentic sample (NSC # 179834) from the NCI pure compound repository.

Glotter E, Abraham A, Gunzberg G, Kirson I. Naturally occurring steroidal lactones with a 17α-oriented side chain. Structure of withanolide E and related compounds. *J. Chem. Soc. Perkin Trans. I.* 1977:341-346.

Kirson I, Abraham A, Sethi P, Subramanian SS, Glotter E. 4β-Hydroxywithanolide E, a new natural steroid with a 17α-oriented side chain. Phytochem 1976;15:340-2.

Lavie D, Kirson I, Glotter E, Rabinovich D, Shakked Z. Crystal and molecular structure of withanolide E, a new steroidal lactone with a 17α-side-chain. J Chem Soc Chem Comm 1972:877-878.

McCloud TG. High throughput extraction of plant, marine, and fungal specimens for preservation of biologically active molecules. Molecules 2010;15:4526-63.

Sakurai K, Ishii K, Kobayashi S, Iwao T. Isolation of 4β-hydroxywithanolide E, a new withanolide from *Physalis peruviana*. Chem Pharm Bull 1976;24:1403-5.

Sensitization of cancer cell lines to TRAIL-induced apoptosis

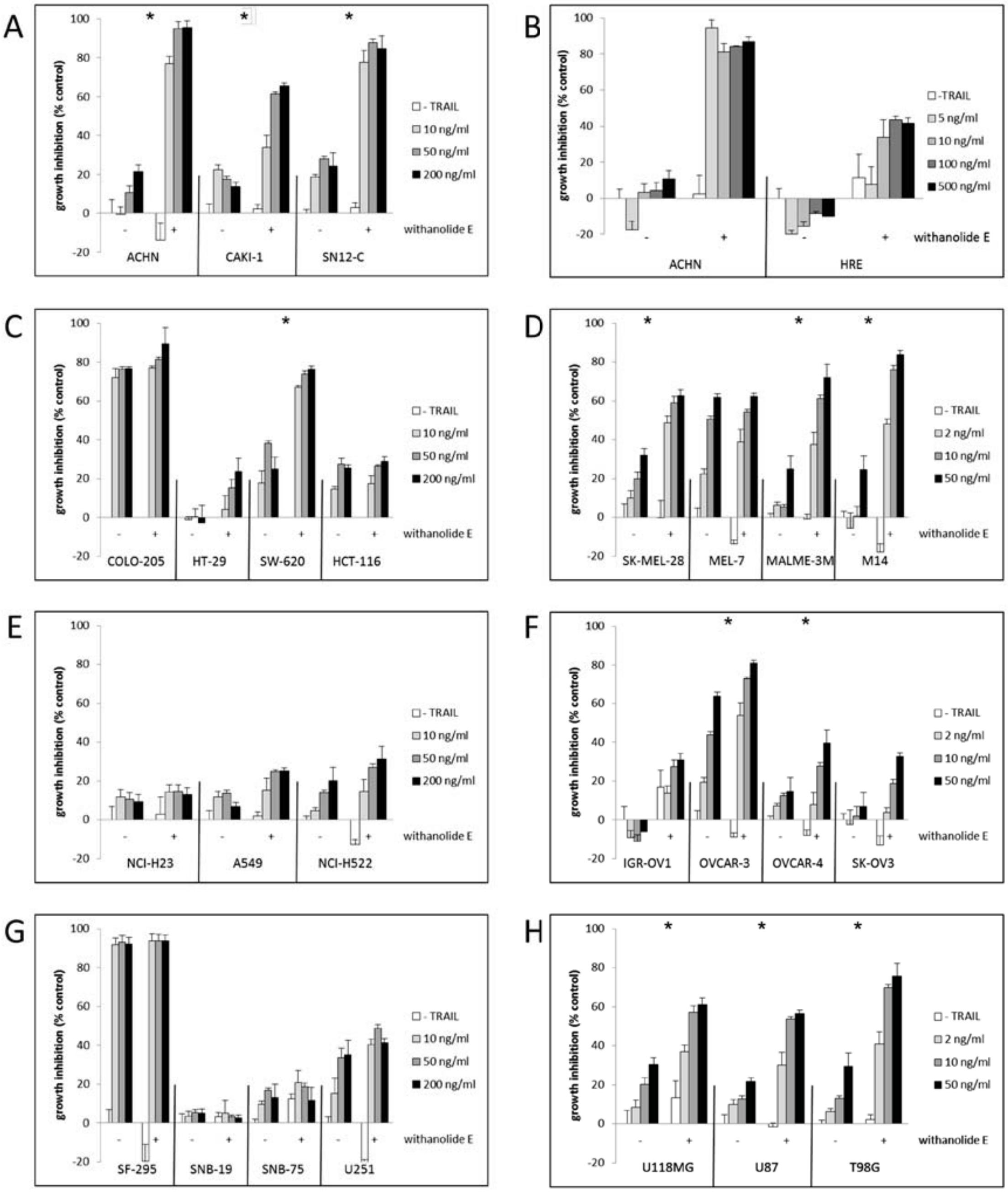

Supplementary Figure 1:

A range of cancer cell lines of various lineages were assessed for sensitization: Panel A, renal; Panel B, in a separate experiment, ACHN and primary human renal epithelial (HRE) cells were compared; Panel C, colon; Panel D, melanoma; Panel E, lung; Panel F, ovarian; Panels G and H, CNS/glioblastoma. Error bars represent sd (n = 3-6). NOTE: Panels A and B recapitulate data from Figure 2 in the main text.

*$p < 0.01$ at two or more TRAIL concentrations comparing TRAIL ± WE (students t-test), *i.e.* TRAIL-resistant becomes TRAIL-sensitive.

ACHN, CAKI-1, SN12-C, COLO-205, HT-29, SW-620, HCT-116, SK-MEL-28, MEL-7, MALME-3M, M14, SF-295, SNB-19, SNB-75, U251, IGR-OV1, OVCAR-3, OVCAR-4, SK-OV3, NCI-H23, A549, and NCI-H522 cell lines were obtained from the National Cancer Institute Developmental Therapeutics Program (Frederick, MD). U87, U118MG and T98G glioblastoma cells were kindly provided by Dr. William Murphy, University of California, Sacramento. Human renal epithelial cells were from Lifeline Cell Technology (Frederick, MD). All cell lines were maintained according to the recommendations of the source institutions. Cells were plated in 96-well cell culture plates at 5000 cells/well. After overnight attachment, cells were treated with 1 μM withanolide E (+) or DMSO control (-) for 4 h followed by indicated concentrations of TRAIL for an additional 24 h (withanolide E remained in the medium throughout the experiment). Cell numbers were estimated by the MTT assay and normalized to DMSO/no TRAIL controls.

Withanolide E has minimal effect on cell morphology

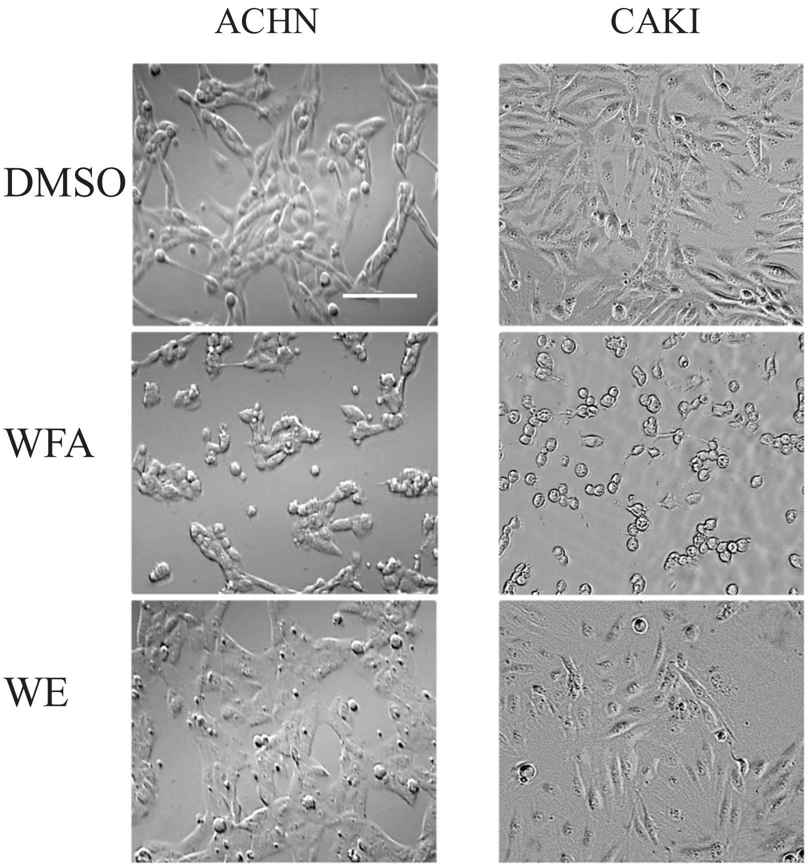

Supplementary Figure 2: ACHN or CAKI cells were seeded at 2 x 10^5 cells/well in 24-well tissue culture treated plates and allowed to attach and recover overnight followed by 8 h treatment with 10 µM withaferin A (WFA), withanolide E (WE), or DMSO control. Photomicrographs were obtained on live cells in growth medium using a Nikon 20X objective (NA 0.45) on a Nikon S2000 microscope with a Nikon Digital DS-Qi1Mc camera. Image acquisition employed Nikon Elements software. Bar indicates 100 µm.

Effects of withanolide E on levels of pro- and anti-apoptotic proteins

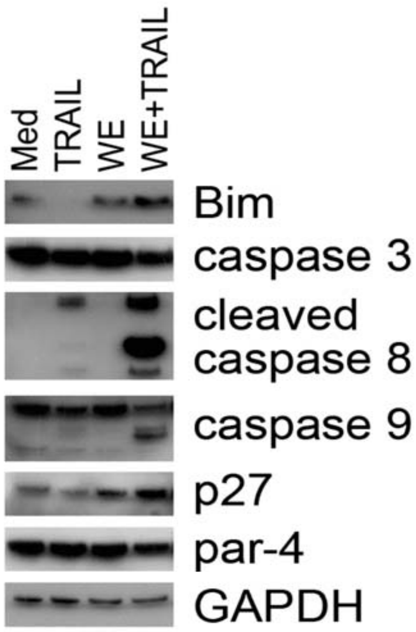

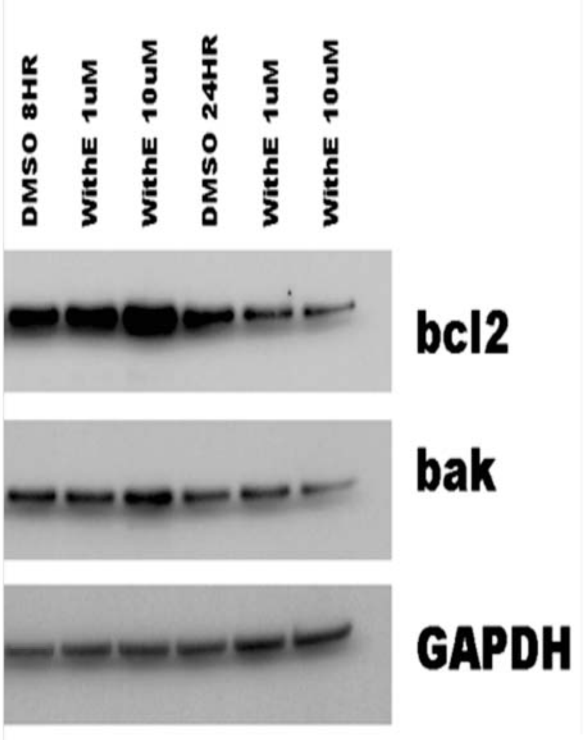

Supplementary Figure 3: Western blot analysis of proteins was performed as described in the Materials and Methods section of the main text. Left panel shows results from ACHN cells treated for 24 h with or without withanolide E (WE, 1 μM) followed by TRAIL for 1 h. Right panel shows analysis of cells treated with withanolide E (WithE) alone.

ACHN cells were seeded in 6-well plates at 2 x 10^6 cells/well. After overnight attachment, compounds were added followed by treatment for varying times after which point cells were lysed (NuPAGE® LDS Sample Buffer and NuPAGE® Reducing Agent, Invitrogen). Proteins were separated under reducing conditions on NuPAGE® 4-12% Bis-Tris gels (Invitrogen), transferred to polyvinylidene difluoride membranes (Invitrogen), probed with primary antibodies followed by appropriate horseradish peroxidase conjugated secondary antibodies and SuperSignal West Femto maximum sensitivity substrate (Thermo Fisher Scientific Inc.).

The following antibodies were employed to provide these results as well as those shown in the main text (Figures 3-6): Antibodies against p27, AKT, pAKT, BAK, BCL2, BCLxl, BIM, caspase 8, caspase 9, CDK2, CDK4, CHOP, cyclin D1, GAPDH, glucocorticoid receptor (GR), and GGRP78 (= BIP) were from Cell Signaling Technology (Danvers, MA). Sources for other antibodies were as follows: cFLIP (Enzo, Farmingdale, NY), FADD and RAF1 (EMD-Millipore, Billerica, MA), cIAP1 (R & D Systems, Minneapolis, MN), p53 (Kamiya Biomedical, Seattle, WA), HSP70 and HSP90 (StressMarq Biosciences, Victoria, BC), BAX, BIK, and PAR4 (Santa Cruz Biotechnology, Dallas, TX). BID, MCL1, XIAP, and p21 antibodies were from B-D Biosciences (San Jose, CA). Appropriate secondary antibodies conjugated with horse radish peroxidase were purchased from Thermo Scientific (Rockford IL).

Effects of withanolide E on expression of cFLIP mRNA:

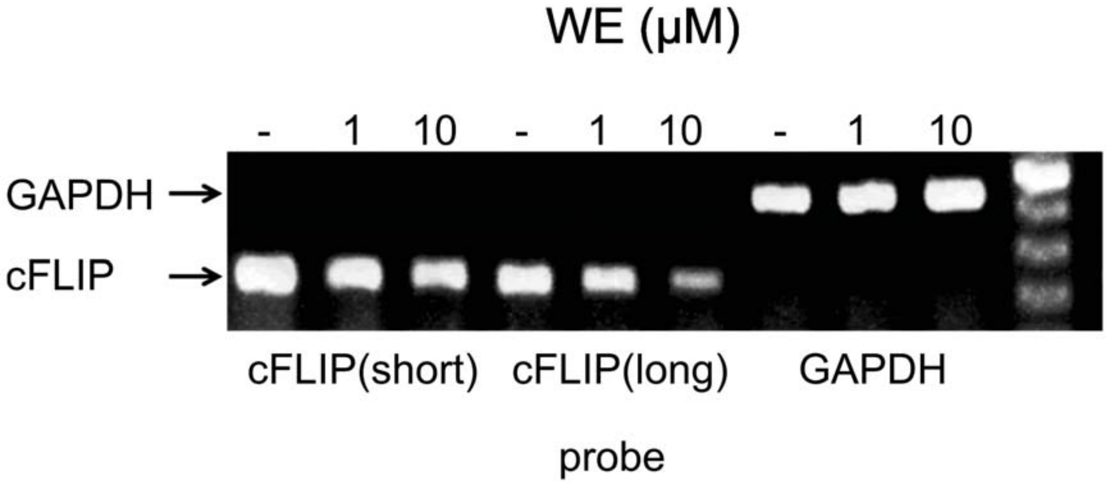

Supplementary Figure 4:
ACHN cells were treated for 8 h with 1 or 10 µM withanolide E (WE).
2 µg of RNA was used to make cDNA using ABI reverse transcription reagents in a 25 µl reaction. 5 µl of cDNA was used for a 50 µl PCR reaction using Invitrogen high fidelity system. PCR conditions were 95 degrees 10 min, 95 degrees 45 sec, 55 degrees 45 sec, 72 degrees 45 sec for 25 cycles. Final extension at 72 degrees for 7 min. The following PCR primers were employed for detection and quantitation of mRNA for cFLIP short and long forms:

cFLIPL_Forward	GGACCTTGTGGTTGAGTTGG
cFLIPL_Reverse	ATCAGGACAATGGGCATAGG
cFLIPs_Forward	GGCTCCCAGAGTGTGTATGG
cFLIPs_Reverse	AGCTTCTCGGTGAACTGTGC

Effects of withanolide E on proteasomal activity

A

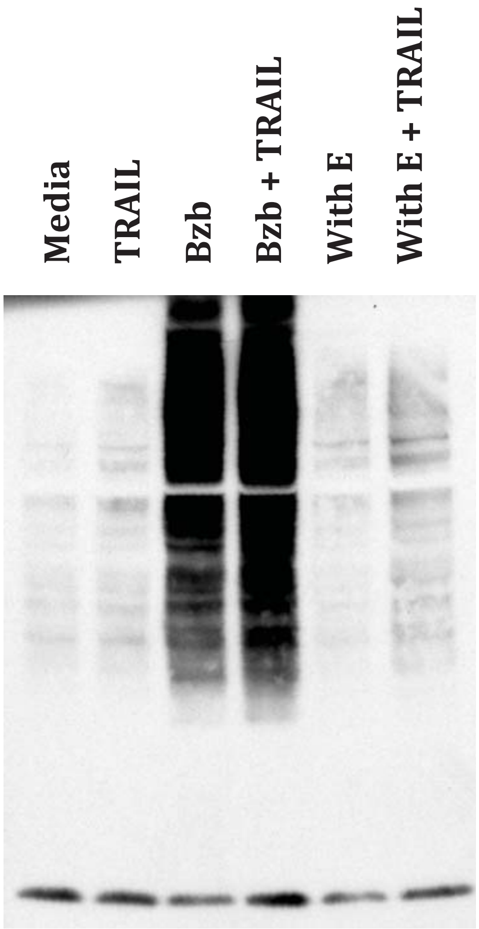

B

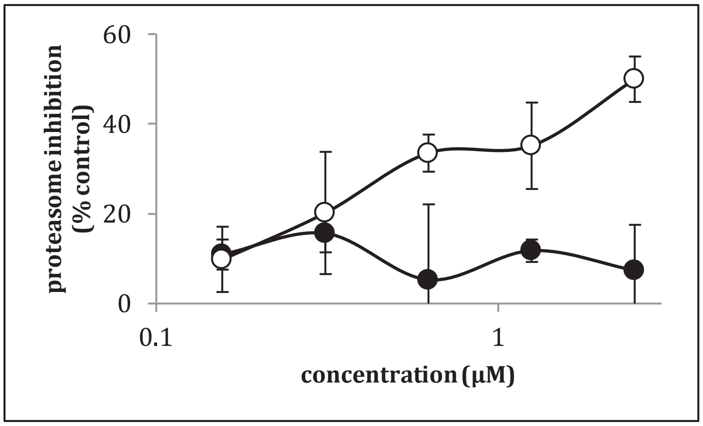

Supplementary Figure 5:
A: ACHN cells were treated for 24 h with 1 µM withanolide E or 40 nM bortezomib and assessed for ubiquitination of cellular proteins by western blot analysis. Total cellular protein was separated by SDS-PAGE and probed in immunoblot using an anti-ubiquitin antibody (P4D1, sc-8017 from Santa Cruz).

B: ACHN ells were treated 8 h with indicated concentrations of withanolide E (black symbols) or withaferin A (positive control – reported in the literature to inhibit the proteasome - open symbols) then proteasomal activity assayed using the Proteasome-Glo Chyotrypsin-Like Cell-Based Assay (Promega). Error bars represent sd (n = 3).

Reaction of withanolide E with N-acetyl cysteine

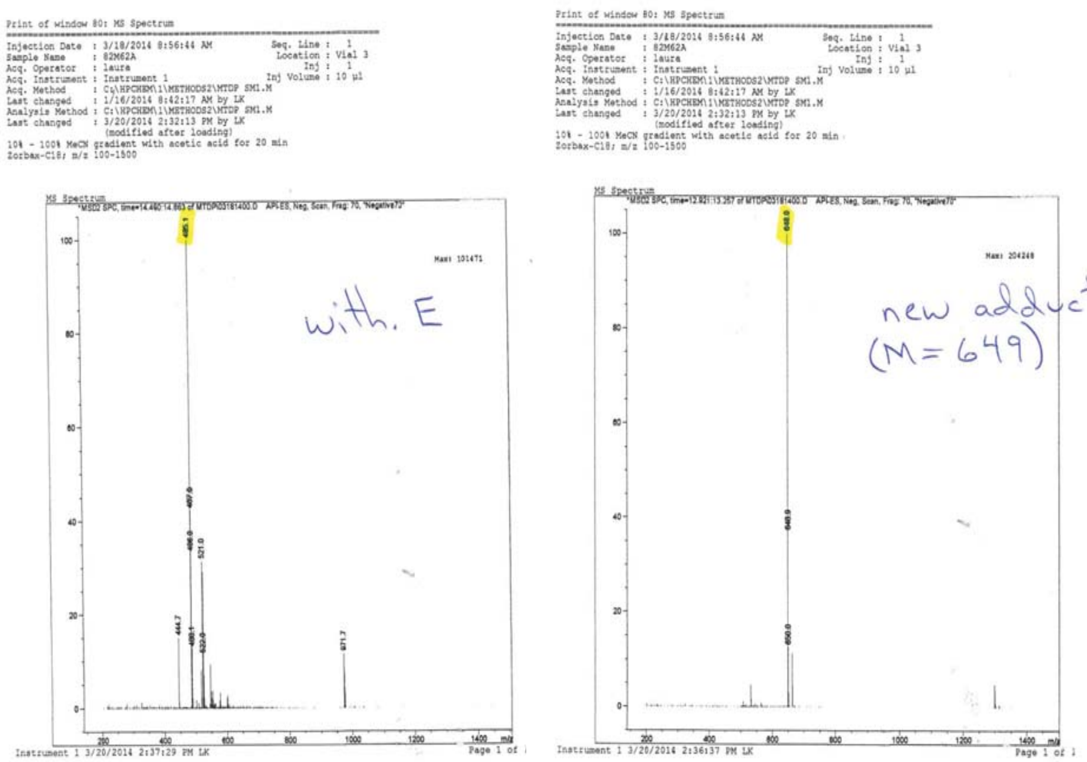

Supplementary Figure 6:
-withanole E (left): Negative ion ESI-MS spectrum of withanolide E observed in LC-MS analysis of the reaction mixture of withanolide E and N-acetyl cysteine (18 hrs).
-NAC-withanolide E adduct (right): Negative ion ESI-MS spectrum of new adduct observed in LC-MS analysis of the reaction mixture of withanolide E and N-acetyl cysteine (18 hrs).

A solution containing a 3:1 mole ratio of NAC and withanolide E in methanol/PBS (pH 8) was analyzed by LC-MS to reveal a covalent aduct of withanolide E-N-acetyl cysteine (mol. wt. 649 da). The ratio of this compound to unreacted withanolide E (mol. wt. 486 da) was 1:2 after 18 h at room temperature. After 42 hrs less than 10% of the original withanolide E remained, additional decomposition products appeared, and the ratio of the components described above was approximately 2:1.
